# Can Smartwatches Replace Smartphones for Posture Tracking?

**DOI:** 10.3390/s151026783

**Published:** 2015-10-22

**Authors:** Bobak Mortazavi, Ebrahim Nemati, Kristina VanderWall, Hector G. Flores-Rodriguez, Jun Yu Jacinta Cai, Jessica Lucier, Arash Naeim, Majid Sarrafzadeh

**Affiliations:** 1 Computer Science Department, University of California, Los Angeles, Los Angeles, CA 90095, USA; E-Mail: majid@cs.ucla.edu; 2 Department of Electrical Engineering, University of California, Los Angeles, Los Angeles, CA 90095, USA; E-Mail:ebrahim@g.ucla.edu; 3 School of Medicine, University of California, Los Angeles, Los Angeles, CA 90095, USA; E-Mails: KVanderWall@mednet.ucla.edu (K.V.W.); jlucier@mednet.ucla.edu (J.L.); ANaeim@mednet.ucla.edu (A.N.); 4 Computer Science Department, El Camino College, Torrance, CA 90506, USA; E-Mail: hct.flr@gmail.com; 5 Computer Science Department, University of Alabama Birmingham, Birmingham, AL 35233, USA; E-Mail: jacintacai@gmail.com

**Keywords:** activity recognition, smartwatch, posture tracking, wireless health, machine learning, embedded medical systems

## Abstract

This paper introduces a human posture tracking platform to identify the human postures of sitting, standing or lying down, based on a smartwatch. This work develops such a system as a proof-of-concept study to investigate a smartwatch’s ability to be used in future remote health monitoring systems and applications. This work validates the smartwatches’ ability to track the posture of users accurately in a laboratory setting while reducing the sampling rate to potentially improve battery life, the first steps in verifying that such a system would work in future clinical settings. The algorithm developed classifies the transitions between three posture states of sitting, standing and lying down, by identifying these transition movements, as well as other movements that might be mistaken for these transitions. The system is trained and developed on a Samsung Galaxy Gear smartwatch, and the algorithm was validated through a leave-one-subject-out cross-validation of 20 subjects. The system can identify the appropriate transitions at only 10 Hz with an F-score of 0.930, indicating its ability to effectively replace smart phones, if needed.

## 1. Introduction

Wearable and mobile sensors are increasingly prevalent, with studies showing users are within proximity of their smartphones almost 90% of the time [1]. These phones have impressive sensing capabilities, ranging from remote health monitoring [2,3] to ubiquitous life-logging [4]. While phones present ample opportunity to track users, studies have shown that, while close to the user, the smartphone often is not actually on the user [5]. Instead, several works suggest wearable sensors and computers might be better suited for human activity recognition applications [6,7,8]. However, the design of the wearable sensor system [9], as well as the methods of interaction [10] lead to important questions about sensing capabilities, as well as interface. The emerging smartwatch market is an extension of such wearable platforms that benefits from being worn by the user, in a standard location, able to track activity, either on its own [11] or along with smartphones [12]. Currently used to track general activity [13] or specialized movements (e.g., weight resistance training) [11], these watches, when fit with the right inertial sensors [14], can continuously track patients similarly to those studies using smartphones, along with providing a programmable user interface. Smartwatches, however, have constraints that may limit their effectiveness, including screen size, weaker hardware for sensing and computing and limited battery and storage capacity [7]. This work investigates whether the smartwatch can effectively track user activity and posture without the aide of a smartphone, to then potentially serve as the base platform for a remote health monitoring system for oncology patients.

Remote health monitoring systems (RHMS) allow for the continuous recording of patient data and identification of patient status in any environment [2,3,15]. These systems range in application, from tracking activity [16,17] to heart failure [18,19,20]. Many accelerometer-based wearable systems have been developed to track human activity [16], weight training [11], exercise intensity [21], to more directed applications, such as fall detection [22]. In particular, interactive devices for healthcare applications are an obvious and necessary application for wearable technology. These systems, and their machine learning algorithms, have the potential to improve patient outcome identification [23], as well as to reduce the cost of intervention and treatment [24].

Elderly patients with cancer are a group that stand to benefit tremendously from remote sensing: they are prone to unwitnessed decline and hospitalization between clinic visits, resulting in high morbidity, mortality and cost. According to the most recent American Cancer Society statistics, 60% of cancers and 70% of cancer deaths occur in adults aged 65 and over. Physicians must make “snap” decisions regarding treatment intensity and followup, but the tools physicians currently use to classify elderly patients as “frail” or “robust” are faulty at best; more than 20% of elderly patients’ cancer doctors classified as fit for therapy are classified as “frail” by geriatric specialists [25]. A full assessment for frailty in an elderly subject can require 45 min or more, which is an impractical time requirement for busy cancer clinics. However, to fully track the posture of these patients, the sensing technology must first be developed and validated for the necessary tracking, on any individuals, before any systems can be developed for actual clinical trials.

This work will investigate the ability of smartwatches to provide the necessary tools to assist in wrist-worn posture tracking in a laboratory setting, as a proof-of-concept study for the use of such a system for clinical assessment. The system will need to track and record the necessary activities and activity levels of users from the wrist rather than the traditional hip locations. In particular, being up and about, sitting or lying in bed is very important for identifying posture and activity in each posture state. However, where smartphones have an advantage of tracking such posture from the hip, a smartwatch should see significant activity in all three phases of posture as a user might move her/his arm while sitting. This paper develops such a smartwatch system, to test whether smartwatches can replace smartphones for posture tracking, rather than simply augment them. This system will need to record activity all day (a goal of about 18 h of battery life) and accurately report patient activity levels. Prior analysis in [26] showed energy consumption in continuous sensing and trade offs with model accuracy. Indeed, by reducing the sampling rate, storing results on the watch and transferring via USB cable rather than via a wireless network and computing results on a host computer, improved battery life should be achievable. The work in [26] then presents the challenge of using smartwatches, after turning off wireless communication and other processes, as the challenge of reducing the sampling rate while maintaining accuracy. This work will investigate the classification accuracy of such a system and provide evidence that a smartwatch, alone, can properly identify the necessary movements to identify human posture without needing a smartphone or other such hip-worn sensor and at a low enough sampling rate to last without needing constant battery recharges or data uploads that might hamper the use of this platform.

## 2. Related Works

### 2.1. Activity Recognition

Activity monitoring with smartphones and devices with these phones have been well studied. Monitoring activities of daily living through wearable sensors or smartphones is highly accurate [17,21]. In particular, the work in [27] looks at activity tracking for a clinical environment and how to guarantee that users are performing the desired activity. This work intends to follow the same model of activity recognition presented there. In particular, by identifying the transitions between sitting, standing and lying and appropriately identifying (and ignoring) all other wrist movements, this work approaches the classification of user posture similarly to the anti-cheating developed in [27]. By showing the same levels of accuracy, this paper will show that smartwatches are capable of replacing smartphones.

### 2.2. Wearable Activity Recognition

The work in [28] investigates the use of wearable activity trackers and the challenges presented. In particular, they evaluate user experience in using the wearable sensor and accuracy of the algorithm to track activity [28]. While this work leaves the user interface for future work, it will focus on validating an accurate posture tracking algorithm to address those challenges presented. The work in [29] uses a wrist-mounted sensor for real-time gesture recognition. This work develops a sensor that can track gestures and reduces power consumption through calibration of the sensor and provides motivation for thinking smartwatches can be used in a configuration to last an entire day. However, they do not present accuracy measurements for their tracking system. The work in [30] uses several wearable sensors in order to accurately detect posture, including the current state (e.g., sitting or standing), as well as the transition between states (e.g., sitting down, standing up) [30]. This paper attempts to replicate the accuracy of the posture tracking presented in [30], but by using only one sensor worn on the wrist. This paper aims to address similar accuracy presented by these works on posture tracking using a smar twatch platform.

### 2.3. Smartwatch Activity Recognition

Smartwatches have been used to provide plenty of activity tracking applications to date [11,31,32]. The work in [11] applied machine learning algorithms to five weight resistance training exercises. It was shown that the watch can provide accurate tracking results similar to a phone or custom sensing environment, as well as provide a custom interface for the application. The work in [31] provided activity tracking for dementia patients. In this case, a tracking and stepping algorithm is developed to monitor patients at risk for falling down and wandering. However, the work uses a centralized server to track the patients’ locations. The work in [32] develops a watch-like sensor to track falls, walking, hand-related shocks and general activity. Using a feature extraction and selection technique, results are presented in a 10-fold cross-validation technique to determine the ability to track elderly patients. This paper extends the methods presented in [11,32], by first finding the appropriate features for proper posture tracking of users with a smartwatch and, second, by doing so with reduced sensing rates to extend the battery life, if possible. The work in [33] presents methods by which continuous measurement on smartwatches can be performed in an energy-efficient manner. While such an intensive energy-expenditure calculation is not conducted in this work, the selection of data and the context of the current state of the user could be used in a similar fashion. For this reason, it is believed that the method presented, along with the reduced sampling rate, would result in the improved battery life of the device. Further analysis of energy expenditure, such as in [33], is left for the limitations and future work discussion in Section 5.2.

## 3. Smartwatch Tracking System

**Figure 1:**
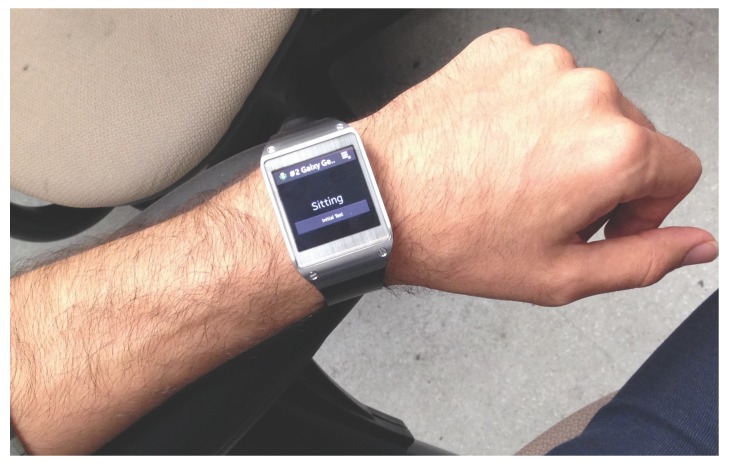
Image of a user wearing the system.

The system developed here was a pervasive sensing system that could be worn by the user at all times, tracking the activity while also prompting questions as needed, seen in Figure 1. The goal of the system was to accurately track activity levels, as well as to provide an interface for important questions necessary for future assessment status, though the interface and its study are left for future work in Section 5.2. This system needed to be able to record and track data for large periods of time in order to provide a more informed classification of a user’s entire day. Further, by identifying the three key posture states of sitting, standing and lying, a classification algorithm is presented that can appropriately identify transition movements of these postures *versus* other activity movements.

### 3.1. Hardware Platform and Data Collection

**Figure 2:**
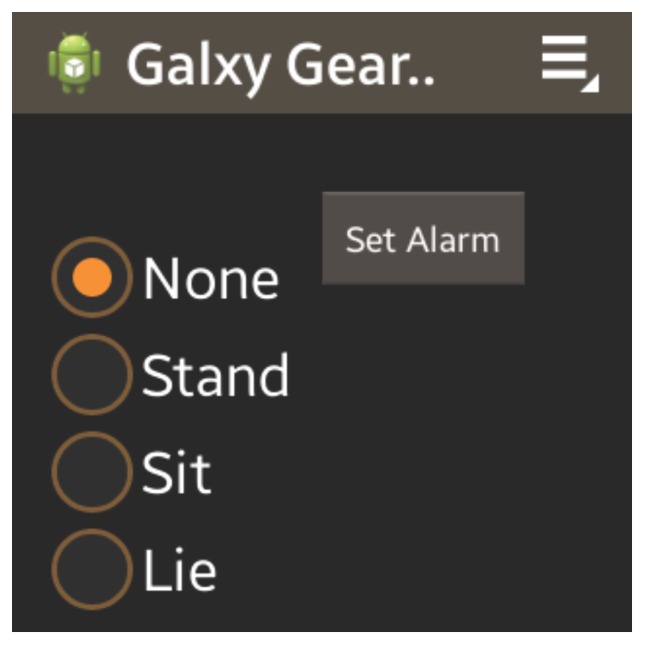
Screenshot of the data-recording application running on the smartwatch.

The Samsung Galaxy Gear smartwatch was used for experimentation, as it employed a ±2-g triaxial accelerometer and a ±300${}^{\circ }$ per second gyroscope sensors, while further providing a software environment for interactive applications and 4 GB of internal storage. Given that the movements recorded are for posture tracking only and the transitions between states tend not to be violent actions, the ±2-g accelerometer was not considered a limitation. Data were stored on the smartwatch in internal memory, which provided ample storage for the duration of the collection desired. All extraneous applications and wireless communication are turned off, and the screen timeout was set to the shortest time possible. Further, the data are uploaded to the host computer via a wired USB cable at the end of each day, and this computer communicates and computes, as necessary. When looking at the watch on the left wrist, the y-axis points to the hand, the x-axis directly up, while the z-axis comes out orthogonal to the watch face. In order to appropriately compare against smartphones, a Samsung Galaxy S4 was used, worn in the pants pocket of the user on the same side that the watch was worn (left) during data collection to simulate hip-worn sensors. The Galaxy S4 smartphone came with a ±2-g triaxial accelerometer and ±300${}^{\circ }$ per second gyroscope sensors, as well, and 2 GB of internal storage data were collected from a group of 20 volunteers within the age bracket of 19–30 years in a supervised study, in order to validate the ability of such a watch to accurately identify such movements. The age group was selected as part of an Institutional Review Board (IRB) approved pilot trial (UCLA IRB #14-000176) to demonstrate the feasibility of such a sensor system. Each of the participants performed multiple activities while wearing a smartwatch placed on the participant’s left wrist and smartphone in their left pocket (though data can easily be transformed to use the right hand, if needed). Data were sampled at a rate of 100 Hz. The data collection application was developed to annotate the data while collecting it, as seen in Figure 2. In order to assist the users and to prevent unrealistic motions of moving the watch to press the buttons, one of the authors supervised the data collection trial and pushed the appropriate buttons for annotation while the users were conducting the collection trial. This was done in an effort to minimize error in the annotation times, as well as to prevent excess movement by the users to begin and end annotations of movements. The annotations were then applied to the smartphone data, as well.

Each subject was then asked to perform a set routine of activities meant to train an algorithm to classify sitting, standing and lying. For each activity, users were asked to repeat each action 10 times. Table 1 shows the list of movements captured. The data were captured in three phases. The first labeled transitions, tracked transitions between sitting in a chair, standing up and lying on a bed (of varying heights depending on the location of the data collection) with little to no extraneous movement. These were the clean movements that the system needed to identify. However, potential trouble in identifying the transitions from a wrist-mounted sensor include the similarity between certain transition movements and certain activities of daily living that can look similar to these transitions. As a result, the second phase of data collection, shown in Table 1 as activities of daily living, was run. Finally, the third phase was run to identify steps for a pedometer-like application. After the data collection was run, the system recorded the user removing the device and plugging it in to the charging environment to properly mark the start and end of a day of recording. Since the movements were annotated, a start and end point for each transition was determined and the mean window size across all users and all moves selected (5 s or 500 points at 100 Hz). Data were saved in the internal storage of both the smartwatch and smartphone. At the end of each day of the trial, data were uploaded manually to a desktop computer for the recognition algorithm, via a USB cable.

### 3.2. Feature Extraction and Selection

The data collected and annotated were then processed for feature extraction. The first step was to low-pass filter the data for noise. For filtering purposes and to potentially coincide with a future real-time recognition system, a moving average window was used. Since the average movement length was 5 s, a movement window of 1 s (100 points) was used, found heuristically to be the best filter. Next, the feature extraction was run. While there are several features that are common in smartphone platforms, including max, min, mean, sum, standard deviation, kurtosis, skewness and energy over a window, because this work incorporated a gyroscope and a different position on the body, a wider range of features were developed from which to be selected. The 25 features, listed in Table 2, were collected for each axis, as well as for the magnitude of acceleration, resulting in 175 total features. These features were selected due to their strengths in various recognition techniques, including activity monitoring, handwriting recognition and wrist-worn tracking [16,17,34,35,36].

**Table 1:** Movements captured.

Phase	Movement State	Activity Description
Transitions	Sit-Stand	Minimal Movement Transition
	Stand-Sit	
	Sit-Lie	
	Lie-Sit	
	Stand-Lie	
	Lie-Stand	
Activities of Daily Living	Standing	Using Phone (10 s)
		Brushing Teeth (10 s)
		Lifting Cup (10 times)
		Swinging Arms(10 times)
		Walk (10 s)
		Open Door (10 times)
		Look at Watch (10 times)
		Clean with Broom (10 s)
	Sitting	Typing (10 s)
		Reading Book (10 s)
		Brushing Teeth (10 s)
		Look at Watch (10 times)
		Bicep Curl (10 times)
		Use TV Remote (10 s)
	Lying	Adjust Pillow (10 s)
		Text with Phone (10 s)
		Adjust in Bed (10 s)
		Reading Book (10 s)
		Adjust Blanket (10 s)
Walk	Step Forward	10 times
	Step Backward	10 times

Once the features were extracted, a selection algorithm was run using Weka’s [37] Information Gain Feature Selection algorithm, with a ranker to provide the top 30 features. The top 30 features were selected to avoid overfitting the model to the training set. In fact, [38] stated that linear support vector machines (SVM) should only have a ratio of 10:1 for features to data samples. In the case of this paper, 20 users repeated each of seven actions (six transitions and a no-movement class). Each repetition of these movements would pollute the 10:1 ratio, so they were not considered in the calculation. As a result, 140 unique data samples were considered, which indicated that 14 features should be the maximum in a linear setting. As this work employed a more advanced kernel, double those features were considered to adhere to the same principal, while accounting for a more advanced kernel and multiclass setting. Once the subset of features was selected, the model was tested in cross-validation for its prediction strength. In particular, it was important to achieve high precision and recall to appropriately identify the transitions between states when performing activities and to avoid identifying false transitions.

**Table 2:** Features extracted per axis.

Feature	Description (Domain)
Minimum	Minimum value obtained over the movement window (time)
Maximum	Maximum value obtained over the movement window (time)
Sum	Sum of values obtained over the movement window (time)
Mean	Mean value obtained over the movement window (time)
Standard Deviation	Standard deviation of values obtained over the movement window (time)
Kurtosis	Peakedness of the distribution (time)
Skewness	Asymmetry of the distribution (time)
Energy	Calculation of the energy (sum of the absolute value of the fftcomponents) (frequency)
Variance	Variance of values obtained over the movement window (time)
Median	Median value obtained over the movement window (time)
Root Mean Square (RMS)	Root mean square of values over the movement window (time)
Average Difference	Average difference of values (pairwise) in window (time)
Interquartile Range	Dispersion of data and elimination of outlier points (time)
Zero Crossing Rate	Rate of sign changes in signal (time)
Mean Crossing Rate	Rate of crossing the mean value of signal (time)
Eigenvalues of Dominant Directions	Corresponds to dominant direction of movement (time)
CAGH	Correlation coefficient of acceleration between gravity and heading directions (time)
Average Mean Intensity	Mean intensity of the signal (time)
Average Rotation Angles	Calculates rotation based on gravity (time)
Dominant Frequency	Dominant frequency in transform (frequency)
Peak Difference	Peak difference of frequencies (frequency)
Peak RMS	Root mean square of peak frequencies (frequency)
Root Sum of Squares	Root sum squares of frequencies (frequency)
First Peak (Energy)	First peak found in energy (frequency)
Second Peak (Energy)	Second peak found in energy (frequency)

### 3.3. Training the Algorithm

In order to classify the motions accurately, Weka’s implementation of a support vector machine (SVM) was used, using the Pearson Universal Kernel (PUK), which is based on the Pearson VII function adapted to a universal kernel. As explained in [39], this kernel function has a remarkable ability to model data well represented by each other commonly found SVM kernel and, as a result, can be considered a universal kernel for learning algorithms. In particular, with enough data, the PUK kernel can be modified, through its optimization of hyperparameters, to look like any other kernel for SVM. The work in [40] applied this kernel to activity recognition, showing higher accuracy than more commonly-used kernels and methods. The parameters were left as default, with the exception of the complexity, which is raised to a value of 100, to further penalize mistakes of the classifier in its optimization routine. The SVM was supplied with training data labeled with eight labels, the labels being the six transition movements, one label for no movement and one encompassing label for all other movements to reduce false positives of the first six labels. The model was tested at 100 Hz, 50 Hz and 10 Hz and compared to the phone in the pocket, as well as combined with the phone to determine the strongest results.

### 3.4. Testing the Algorithm

The recognition algorithm was then validated to ensure the proper development of a system to accurately track the posture of users. While the activity level was presented as a general magnitude of acceleration, the state of the user provided context to the level of activity achieved and the duration of those activities. As a result, the algorithm needed to be strongest at determining the posture of a user.

#### Cross-Validation

A leave-one-subject-out cross-validation (LOSOCV) was used to determine the model’s effectiveness over user populations. This was chosen over a commonly-used 10-fold cross-validation because of the potential for the pollution of the results in training and testing on the same user. In this manner, it became possible to interpret results as they extend to new users not in the training system. Each move of the test subject was feature extracted and tested. For each movement, a label is known for the ground truth transition state. If the algorithm appropriately classifies the movement with the appropriate class, then this is considered a true positive result. If not, it is considered a false negative result. For example, if the user is standing and sits in a chair, this should be a stand-to-sit transition. If this movement is appropriately classified as a stand-to-sit, it is considered a match and a true positive. If, however, the system calculates this movement as stand-to-lie, then this movement counts as a false negative for stand-to-sit and a false positive for stand-to-lie. From these true positive, true negative, false positive and false negative results, precision and recall are derived, per class. We then average these results for the precision and recall of the system. This micro-averaging result does not bias toward a specific movement, since the quantities of each label are equal. Further explanation of this can be found in [41]. The results for this study were presented by reporting the F-score of each test subject and then averaging those F-scores. The F-score, sometimes referred to as the micro f1 score, is:

(1) $$F=2\times \frac{P\times R}{P+R}$$

where *P* is the precision of the system and *R* is the recall (also known as the sensitivity). Thus, the F-score is an indication of how well the system can identify the transition movements. The F-score is used as a measurement to better indicate the ability of an algorithm to detect all movements and to reduce false positives at the same time, often a more reliable measurement of performance than accuracy.

## 4. Results

### 4.1. Experimental Setup

A leave-one-subject-out cross-validation was run on all subjects in the training set (20). The first step was the feature extraction, then the validation that the smartwatch can accurately classify the movements necessary at only 10 Hz. Then, these results were compared to 50 Hz and 100 Hz in order to compare the differences, along with comparing the phone in the pocket, and using both datasets together. Further, battery usage was compared, as well. In order to compare the battery life of each system, the data collection platform was run until the watch died, with timestamps of the outputted data providing durations. The 10-Hz smartwatch collected, on average, about 19 h of data, while the 50-Hz version lasted only 9 h and the 100-Hz version only about four hours. The large discrepancy is due likely not only to the sensor usage, but power associated with the storage of larger files of more data points. Data were logged and stored on the smartwatch and were under the 4 GB of internal storage per day. For the purposes of this work, this validates the use of only 10-Hz data and that it can provide the necessary duration and store the necessary data for offline communication and computation. Further energy analysis in actual use is left for discussion in Section 5.2. The PUK kernel was also compared against a radial basis function (RBF) kernel, which was more commonly found in activity recognition systems, to validate the selection of the chosen kernel, as well as a commonly-used method with that kernel used for recognition of activities of daily living (ADL) [16,17].

### 4.2. Summary View

**Figure 3:**
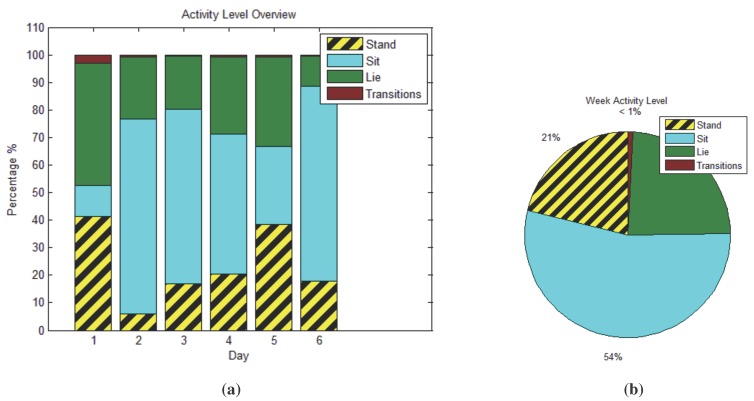
Clinician summary view of the weekly activity of a user from the trial. (**a**) Daily transition and state information of a user from the trial; (**b**) summary of the week.

Figure 3 shows a summary view of the data collected by the system over six days for one of the user’s from the collected IRB trial. Figure 3a show the daily breakdown, while Figure 3b shows the total for a week. The accuracy of this view is dependent on the accuracy of each individual movement recognized, discussed below and further in Section 5.2.

### 4.3. Feature Selection

The top features for the method run at 10 Hz are presented in Table 3. The reason only 10 Hz is shown is because of its success at saving battery life and accurately determining the user state, though, for the watch, the 50-Hz and 100-Hz methods result in similar feature sets. Incidentally, the comparison to the smartwatch and smartphone data used shows that the combination uses primarily watch features (only four phone features at 10 Hz).

**Table 3:** Top 30 features selected for the smartwatch at 10 Hz (and the axis).

Features 1–10	11–20	21–30
Average Difference ($a_{x}$)	Mean ($g_{y}$)	Mean ($a_{x}$)
Average Difference ($a_{z}$)	Sum ($g_{y}$)	Sum ($a_{x}$)
Median of Intensity of Gyroscope (∥g∥)	Eigenvalues ($a_{x}$)	Dominant Frequency ($g_{x}$)
Mean ($g_{z}$)	Root Mean Square ($a_{x}$)	Energy ($g_{x}$)
Sum ($g_{z}$)	Energy ($a_{x}$)	Root Mean Square($g_{x}$)
Dominant Frequency ($g_{z}$)	Root Sum of Squares ($a_{x}$)	Root Sum of Squares ($g_{x}$)
Energy ($g_{z}$)	Standard Deviation ($g_{z}$)	Peak Difference ($g_{y}$)
Root Sum of Squares ($g_{z}$)	Variance ($g_{z}$)	Peak Difference ($g_{x}$)
Root Mean Square ($g_{z}$)	Variance ($g_{x}$)	Dominant Frequency ($g_{y}$)
Peak Difference ($g_{z}$)	Standard Deviation ($g_{x}$)	First Peak ($g_{z}$)

### 4.4. Cross-Validation Results

**Figure 4:**
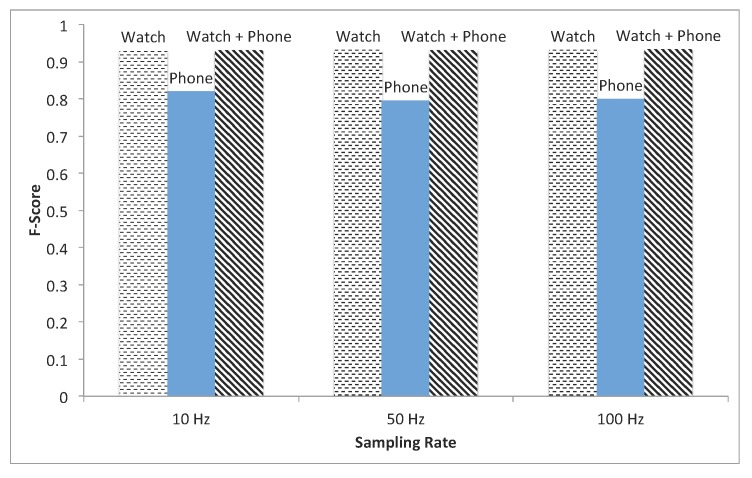
F-scores at three sampling rates for the watch, phone and watch + phone.

**Figure 5:**
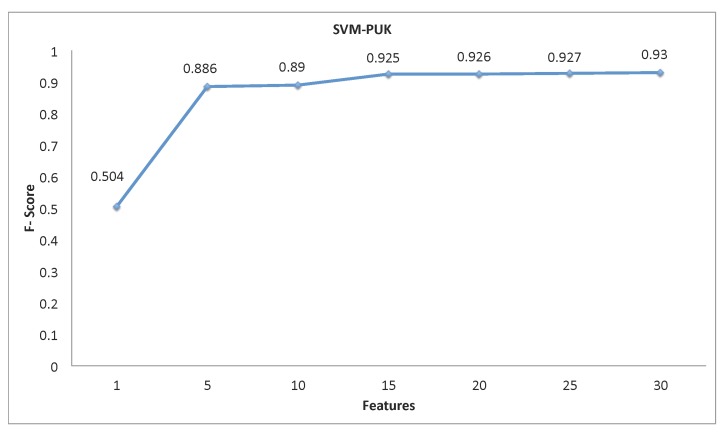
F-score per features used for the smartwatch at 10 Hz with a Support Vector Machine (SVM) using a Pearson Universal Kernel (PUK).

The cross-validation was run in three cases, using data from the watch only, using data from the phone only and using the data from both the watch and phone together, and at the three sampling rates, as discussed: 10, 50 and 100 Hz. Results are plotted in Figure 4. For the 10-Hz case, the algorithm using only data from the watch achieves a mean F-score of 0.93, using data only from the phone a mean F-score of 0.82 and using data from the watch and phone a mean F-score of 0.94. For the 50-Hz case, the algorithm using only data from the watch achieves a mean F-score of 0.93, using data only from the phone a mean F-score of 0.80 and using data from the watch and phone a mean F-score of 0.94. For the 100-Hz case, the algorithm using only data from the watch achieves a mean F-score of 0.93, using data only from the phone a mean F-score of 0.80 and using data from the watch and phone a mean F-score of 0.94. Finally, when selecting a subset of features by rank, Figure 5 showed a high mean F-score with only the top 15 features.

### 4.5. Comparison of Methods

The algorithm, using data sampled at 10 Hz, was compared against the same method using an RBF kernel instead, as well as the ADL algorithm using an RBF kernel. The ADL algorithm used the three-axis accelerometer attached to the hip and extracted features over a 5-s window of the mean, standard deviation, energy and correlation of each axis of the accelerometer [17]. This same algorithm was also extended to use the gyroscope. The phone version was labeled pADL and was also run on the watch data, labeled wADL. Results for all of the methods are in Table 4.

**Table 4:** F-scores of SVM with PUK, SVM with RBF, activity of daily living (ADL) Algorithm [17] and the ADL algorithm with the gyroscope (all at 10 Hz) (phone then watch).

Algorithm	F-Score
SVM (PUK)	0.930
SVM (RBF)	0.812
pADL (AccelOnly)	0.702
pADL (Accel + Gyro)	0.783
wADL (Accel Only)	0.814
wADL (Accel + Gyro)	0.908

## 5. Discussion

### 5.1. Review of the Results

The results of the experiments show promise in using the smartwatch as the platform of choice for future remote health monitoring applications. The first important finding is that the watch, on its own, can accurately detect posture and transitions between postures. The watch alone provides strong results, with an F-score of 0.930 at only 10 Hz compared to 0.932 at 100 Hz. This indicates that the system can, in fact, provide strong classifications of the transition movements and, as a result, determine the state of the user at all times, while also using minimal internal storage and minimal battery life. Further, the watch, in fact, outperforms the phone in this particular method. The reasoning for this seems to be because the phone does a poor job at identifying the general activities, since the hip is relatively still during those, and *versus* no movement at all.

In regards to the 10-Hz watch method, the sensor is particularly suited for this application. Referring once again to Table 3, notice the strength of the gyroscope features, indicating its importance in such systems. Further, the response to the algorithm and the features is shown in Figure 5. The maximum comes at 30 features, but if the strict 14 features of the linear SVM are desired, the system still produces an F-score of 0.925.

Finally, such platforms must come with independent machine learning algorithms tailored to their usage. When examining the results in Table 4, it becomes clear that generally, strong methods of recognition for activities of daily living do not perform as well when tailored to specific applications. Note, in fact, that the ADL algorithm was extended to the watch and with the gyroscope and provides an F-score of 0.91, which is quite good, but can be improved when picking specific features trained for the specific dataset and movements required.

### 5.2. Limitations and Future Work

While the results are strong and the system demonstrated can determine the posture of a user, there are several questions that can still be addressed. The system, as developed, is trained on younger users performing the actions in a laboratory setting. The method of supervision provided likely results in users performing the actions in a fixed way, despite attempts at incorporating variations into the data. When expanded to real-world environments, the types of movements and the speed with which they are performed will likely vary even further. In order to address this, The next step on the movement algorithm is to extend this to the remainder of the IRB trial and to track user’s over a duration of a week for accuracy in a non-laboratory setting and battery usage, in essence, comparing the weekly log results with the user information. The second is to develop the qualitative aspect of the future remote health monitoring system aimed at tracking elderly cancer patients. This phase intends to take advantage of the programmable user interface to present qualitative questions regarding symptoms and general health to augment the quantitative information developed here. This includes running a full user-experience trial on patients in a clinical trial in order to determine the effectiveness of such a system, particularly in comparison to one with a phone, and to determine if the small screen poses a difficult challenge to the elderly patients, all once the watch algorithm has been validated to effectively track in real-world environments. The third question that arises is the complexities of energy efficiency. In the presented work, the smartwatch is limited to a basic logging application and interface or a real-time tracking application. The real-time tracking application does not meet the energy standards necessary, and this work validates only the smartwatch as an appropriate logging tool for offline computation. Understanding online computation, or even keeping this application offline, but allowing for the other standard uses of the smartwatch in real environments will likely effect the battery usage, and as such, a more complex analysis of the energy will be necessary, as conducted in [33].

In particular a patient frailty assessment value developed by the Eastern Cooperative Oncology Group (ECOG) would be a good benchmark for such a system. ECOG is a measurement of oncology patient frailty, shown in Table 5. In order to develop a stronger patient ECOG assessment tool, this platform should be extended to test on elderly patients and over a larger duration of time to better validate the effectiveness of such tools. Such a trial has already begun, approved by the UCLA Institutional Review Board (IRB #14-000914). A refined development of the activity level can also be investigated to give a more quantitative binning in relation to elderly patients specifically, but this might need to be based on perceived ECOG status and account for varying levels of activity for each age group. Further, ECOG status itself will require its own clinical trial, recording on patients of all functional status, to eventually develop a system that gives the indication of ECOG status along with the quantitative and qualitative information presented to clinicians to help with such decision making.

**Table 5:** Eastern Cooperative Oncology Group (ECOG) definitions.

ECOG Value	ECOG Description
0	Fully active, able to carry on all pre-disease performance without restriction
1	Restricted in physically strenuous activity, but ambulatory and able to carry out work of a light or sedentary nature
2	Ambulatory and capable of all self-care, but unable to carry out any work activities. Up and about more than 50% of waking hours.
3	Capable of only limited self-care, confined to bed or chair more than 50% of waking hours.
4	Completely disabled. Cannot carry out self-care. Totally confined to bed or chair.

### 5.3. Conclusions

This work introduced a smartwatch-based system to assist in tracking the posture of users wearing a wrist-worn platform instead of a hip-worn platform. This paper investigated the feasibility of developing such a system to be worn all day (removed only for charging at night). A platform was developed based on the Samsung Galaxy Gear, which allows activity tracking and the execution of custom Android applications. This system allowed for the collection of a week of activity to track the posture of the user. In fact, the recognition results of an F-score of 0.930 for the watch running at only 10 Hz is a promising result for a watch-only system to monitor human posture. Further, the features selected were presented to guide the future development of smartwatch applications, an emerging field with the advent of ever-increasingly powerful wrist-wearable devices. The work presented shows the capabilities of such a device in tracking human posture, enabling future development and trials in more complex environments and with varied user populations.
